# The cGAS-STING Pathway: Novel Perspectives in Liver Diseases

**DOI:** 10.3389/fimmu.2021.682736

**Published:** 2021-04-29

**Authors:** Dongwei Xu, Yizhu Tian, Qiang Xia, Bibo Ke

**Affiliations:** ^1^ The Dumont-UCLA Transplant Center, Division of Liver and Pancreas Transplantation, Department of Surgery, David Geffen School of Medicine at University of California, Los Angeles, Los Angeles, CA, United States; ^2^ Department of Liver Surgery, Renji Hospital, Shanghai Jiaotong University School of Medicine, Shanghai, China

**Keywords:** DNA sensor, cyclic GMPAMP synthase, stimulator of interferon genes, Innate immunity, inflammation, liver diseases

## Abstract

Liver diseases represent a major global health burden accounting for approximately 2 million deaths per year worldwide. The liver functions as a primary immune organ that is largely enriched with various innate immune cells, including macrophages, dendritic cells, neutrophils, NK cells, and NKT cells. Activation of these cells orchestrates the innate immune response and initiates liver inflammation in response to the danger signal from pathogens or injured cells and tissues. The cyclic GMP-AMP synthase (cGAS)-stimulator of interferon genes (STING) pathway is a crucial signaling cascade of the innate immune system activated by cytosol DNA. Recognizing DNA as an immune-stimulatory molecule is an evolutionarily preserved mechanism in initiating rapid innate immune responses against microbial pathogens. The cGAS is a cytosolic DNA sensor eliciting robust immunity *via* the production of cyclic GMP-AMPs that bind and activate STING. Although the cGAS-STING pathway has been previously considered to have essential roles in innate immunity and host defense, recent advances have extended the role of the cGAS-STING pathway to liver diseases. Emerging evidence indicates that overactivation of cGAS-STING may contribute to the development of liver disorders, implying that the cGAS-STING pathway is a promising therapeutic target. Here, we review and discuss the role of the cGAS-STING DNA-sensing signaling pathway in a variety of liver diseases, including viral hepatitis, nonalcoholic fatty liver disease (NAFLD), alcoholic liver disease (ALD), primary hepatocellular cancer (HCC), and hepatic ischemia-reperfusion injury (IRI), with highlights on currently available therapeutic options.

## Introduction

Liver disease presents a globally recognized health threat with a mortality rate of 2 million deaths per year worldwide (1). It often occurs in response to hepatocyte injury caused mainly by the hepatitis B and C virus, alcohol abuse, bile duct damage, nonalcoholic fatty liver disease (NAFLD) or nonalcoholic steatohepatitis (NASH) (2–4). Hepatic inflammation is a critical player in triggering liver diseases. During the initial event of hepatic inflammation, innate immune cells, such as macrophages, neutrophils, natural killer (NK) cells, and NKT cells recognize cell damage or invading pathogens with intracellular-expressed pattern recognition receptors (PRRs) at the cell surface. PRRs detect distinct evolutionarily conserved structures on pathogens, termed pathogen-associated molecular patterns (PAMPs), and trigger innate inflammatory responses by activating a multitude of intracellular signaling pathways (5). Indeed, the innate immune system depends on PRRs, including the cyclic GMP-AMP (cGAMP) synthase (cGAS) and its downstream effector stimulator of interferon genes (STING), inflammasomes, and Toll-like receptors (TLRs) that recognize PAMPs and coordinate antimicrobial defense (6–9). PRRs also recognize a plethora of damage-associated molecular patterns (DAMPs), such as nucleic acids of uncontrolled death of host cells, to further activate the innate immune system, contributing to inflammatory diseases and cancer (10, 11). Therefore, aberrant nucleic acid recognition has emerged as a critical host defense mechanism mediated by cytosolic nucleic acid sensors.

DNA generally resides within the nucleus and mitochondria of eukaryotic cells. Aberrant presence of DNA in the cytoplasm from cellular damage or infection elicits robust immunity leading to activation of type I interferon-stimulated genes (ISGs) that confer increased susceptibility to the pathogens and promote host survival (12). The most robust response following DNA stimulation is initiated by cyclic GMP-AMP (cGAMP) synthase (cGAS), which is activated upon binding to double-stranded DNA (dsDNA) (13). cGAS is a critical cytosolic DNA sensor that catalyzes the synthesis of cGAMP from ATP and GTP and activates type I interferons (IFNs) through the endoplasmic reticulum (ER)-resident adaptor protein STING (13, 14), which subsequently activates the transcription factors NF-kB and IFN regulatory factor (IRF) 3 *via* the TANK-binding kinase 1 (TBK1) (13, 14). Besides, the binding of cGAS to DNA is irrespective of DNA sequence (15). Thus, self-DNA from the mitochondria or nucleus could also act as the cGAS ligand to activate the cGAS-STING pathway in triggering inflammatory responses (16). Recent studies suggested that endogenous cGAS was tightly tethered in the nucleus and prevented its autoreactivity against self-DNA (17–19). The structural basis for inhibiting cGAS by chromatin was verified *via* cryo-electron microscopy by other studies (20, 21). Moreover, cGAS was reported to inhibit homologous recombination-mediated DNA repair and promote genome destabilization, micronucleus generation, and cell death under conditions of genomic stress *via* a STING-independent manner (18). These findings indicate that activation of the cGAS-STING pathway by exogenous or endogenous DNA may contribute to the development of various human diseases. Here, we provide an overview of the cGAS-STING pathway in immunity. Moreover, we summarize and discuss the role of the cGAS-STING DNA pathway in a variety of liver diseases. Finally, we highlight current or prospective therapeutic strategies targeting the pathway.

## Activation of the cGAS-STING Pathway

**Figure 1:**
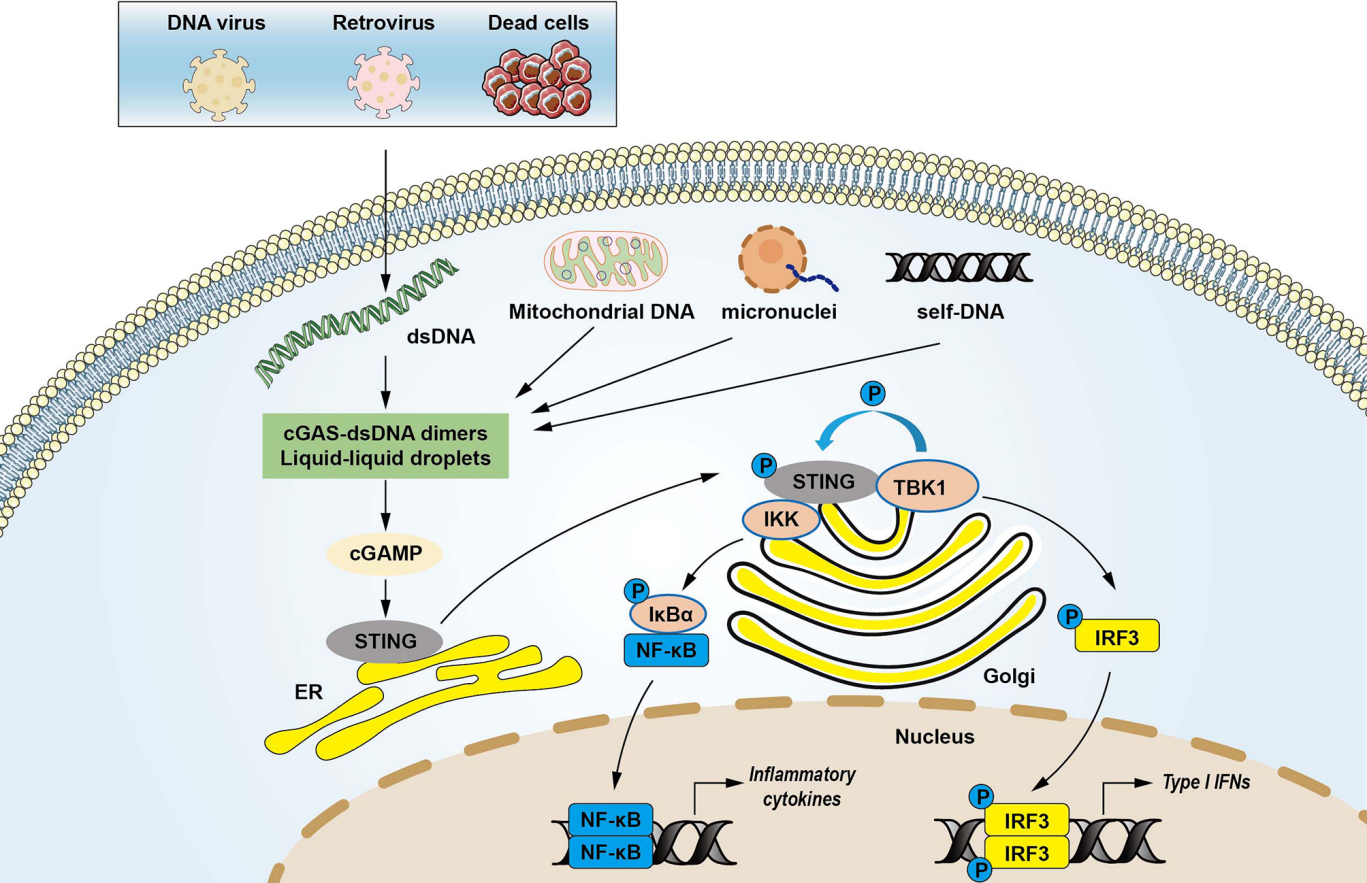
The cytosolic DNA-sensing cGAS-STING pathway in innate immunity. Cyclic GMP-AMP synthase (cGAS) is a protein, which detects various cytosolic dsDNA, including viral DNA, damaged self-DNA released by dying cells, micronuclei, and mitochondrial origins. dsDNA activates cGAS *via* forming cGAS-dsDNA in 2:2 complexes. Mitochondrial damage and the release of mitochondrial DNA (mtDNA) in the cytosol also activates cGAS. The interactions of cGAS with DNA induce the formation of the liquid droplets through a phase transition, in which cGAS exerts its catalytic role to create the second messenger cGAMP that stimulates the stimulator of interferon genes (STING) at the endoplasmic reticulum (ER). STING then translocates from the ER to Golgi compartments and recruits kinases such as TANK-binding kinase 1 (TBK1) and IκB kinase (IKK), which in turn catalyzes the phosphorylation of IFN regulatory factor 3 (IRF3) and the nuclear factor-κB (NF-κB) inhibitor IκBα. Phosphorylated IRF3 translocates to the nucleus to activate transcription of genes encoding type I interferons and other inflammatory genes. IκBα phosphorylation accelerates nucleus translocation of NF-κB to promote transcription of target inflammatory cytokines, leading to activating inflammatory responses.

DNA is a crucial DAMP that is recognized by innate immune receptors and triggers intracellular signaling cascades  (22). dsDNA is primed by damaged mitochondria, dying cells, DNA damage, genomic instability, bacteria, DNA viruses, and retroviruses (12, 23, 24). DNA viruses can induce type I interferon production through activation of the STING pathway (25). Emerging evidence demonstrated that cGAS was required to trigger innate immune response during HIV and other retrovirus infections (26). The cGAS consists of a critical catalytic domain, C-terminal nucleotidyltransferase (NTase) domain, which is composed of two structural lobes with the active site (7). dsDNA activates cGAS by forming 2:2 cGAS-dsDNA complexes (27, 28). The stabilized structure modulates the catalytic domain’s rearrangement to transform GTP and ATP to cGAMP through induction of a conformational change in the C-terminal domain (13, 27, 28). cGAMP is an endogenous second messenger with a high affinity for STING (29). The binding of cGAMP to STING promotes STING translocation to the Golgi apparatus and activates TBK1, which phosphorylates STING and IRF3 transcription factor (13). The activated IRF3 enters the nucleus and triggers the production of type I IFNs, leading to the expression of IFN-stimulated genes (7, 30). STING can also recruit IκB kinase (IKK), which in turn catalyzes the phosphorylation of the nuclear factor-κB (NF-κB) inhibitor IκBα. IκBα phosphorylation accelerates nucleus translocation of NF-κB to promote transcription of target inflammatory cytokines (16). In addition, the N-terminal domain is also responsible for the maintenance of the liquid phase dsDNA and cGAS (31, 32). DNA binding to cGAS promotes the formation of liquid-like droplets, which facilitates cGAS activation *via* augmented cGAS liquid phase separation and enzyme activity (31). These findings demonstrate the multivalent interactions between DNA and the binding domain of cGAS in activating innate immune signaling (**Figure 1**).

## The cGAS-STING Pathway in Viral Hepatitis

Hepatitis B virus (HBV) and Hepatitis C virus (HCV) infections remains a major public health problem in the 21^st^ century with over 300 million people worldwide affected, despite the implementation of various therapeutics (33, 34). HBV is an enveloped partially double-stranded DNA virus (35). HBV infection of human hepatocytes leads to acute and chronic hepatitis, which remarkably increases the risk of liver cirrhosis and hepatocellular carcinoma (HCC) (33, 36). The role of innate immune response in the HBV natural infection process remains unclear and controversial (37, 38). Accumulating data suggested that HBV can escape from recognition by the innate system (39–42). Lacking strong innate immune responses may also account for the convenient transformation of HBV infections to chronic HBV hepatitis (43). Other studies have identified that HBV-derived dsDNA fragments (44), viral genomic relaxed circular (RC) DNA (45), and naked HBV genome (46) could activate the innate antiviral immune responses. As a critical DNA cytosolic DNA sensor, the role of the cGAS-STING pathway during HBV infection has been investigated by several research groups (42, 44, 46–48). Recent studies demonstrated that both primary murine hepatocytes and primary human hepatocytes (PHH) failed to produce type I IFN in response to the foreign DNA in the cytosol or HBV infection due to the lack of STING expression in these hepatocytes (42). However, the hepatoma cell line HepG2 showed an innate immune response after HBV infection since STING expression was observed (42). The lack of DNA-sensing signaling impaired the hepatocytes’ ability to control HBV but induction of STING *in vivo* reduced viral gene expression and replication in hepatocytes (42), suggesting that the absence of the intracellular DNA-sensing pathway dampens the innate immune response against HBV infection in hepatocytes. These results were further validated by another *in vitro* study, which showed that increased STING expression exhibited resistance to HBV infection whereas disruption of STING expression depressed IFN response and enhanced HBV transcription activity in human immortalized hepatocyte NKNT-3 cells (49). Thus, the STING pathway is essential for modulating susceptibility to HBV.

Interestingly, another study suggested that the packaged HBV genome evaded recognition by innate immune cells during natural infection, while naked HBV genomic rcDNA was sensed in a cGAS-dependent manner in human hepatoma cell line HepG2-NTCP (46). Moreover, HBV infection could inhibit the cGAS expression and function in cell culture and humanized liver chimeric mice by downregulating the cGAS-related gene MB21D1, a classic member of IFN-stimulated genes (ISGs) (46). However, HBV-derived dsDNA can also induce the innate immune response by expressing high levels of cGAS in human hepatoma Li23 cells (44). Activation of the cGAS-STING pathway induced ISG56, one of the antiviral genes mediated by type I IFNs, and inhibited HBV assembly (44). Moreover, activation of the cGAS-STING pathway by dsDNA or cGAMP significantly depressed HBV replication *in vitro* and *in vivo* (48). A recent study revealed that HBV DNAs but not RNAs in the viral particles were immunostimulatory and sensed by the cGAS-STING pathway in HepG2 cells (47). HBV rcDNA triggered the hepatocyte response, whereas HBV infection did not suppress the DNA-sensing pathway but can evade the surveillance of the cGAS-STING mediated immune response (47). Indeed, activation of cGAS or STING with pharmaceutical treatment induced IFN response and inhibited viral replication in HBV-infected human hepatoma cells and immortalized mouse hepatocytes (50, 51). As an essential part of the innate immune system, Kupffer cells, which are macrophages residing in the liver, may also contribute to detecting foreign DNA and induction of inflammatory response by phagocytosis during HBV infection. Unlike PHH, the Kupffer cells certainly have intact DNA-sensor signaling, as they exhibit significantly enhanced cGAS-STING pathway levels after HBV infection (41, 42). Pharmaceutical activation of STING by 5,6-dimethylxanthenone-4-acetic acid (DMXAA) in macrophages could remarkably inhibit hepatocyte HBV replication in mice (50). Although Kupffer cells are positive regulators of antiviral immunity during HBV infection (37), the HBV core has been known to activate TLR2 on Kupffer cells leading to inhibition of HBV-specific T cell response by producing IL-10 (52). Genetic knocking out of TLR2 or pharmaceutical depletion of Kupffer cells resulted in a stronger antiviral immune response (52). Another study suggested that instead of promoting liver inflammation, Kupffer cells can inhibit immune response by removing apoptotic hepatocytes during HBV infection (53). These conflicting results on the role of the cGAS-STING pathway in hepatocytes and Kupffer cells during HBV infection suggest that more investigation is needed to clarify the underlying mechanism. Thus, it would be interesting to explore the cGAS-STING signaling role in other innate immune cell types in HBV infection.

HCV infection, followed by liver failure, liver cirrhosis, and HCC, is considered one of the most common causes of liver transplantation in Western countries (54). Evading innate and adaptive immune responses is the primary mechanism for HCV to defeat host immune surveillance and responses. The mechanism underlying HCV regulaton of host interferon response has been investigated for years. Several studies revealed that casein kinase II (CK2) was required for HCV core protein-mediated modulation (55) and served as a critical regulator in controlling IFN response. Activation of CK2 inhibited retinoic acid-inducible gene I (RIG-I)-mediated immune response, whereas disruption of CK2 promoted STING-mediated TBK1 activation and triggered IFN-β immune defense against HCV infection (56, 57). In addition, the hepatitis C virus non-structural 4B (HCV-NS4B) protein, an essential component of viral replication, was found to directly and specifically bind to STING and block the STING-Cardif interaction, contributing to potent inhibition of RIG-I-medicated IRF-3 phosphorylation and IFN-β (58). HCV-NS4B was also found to impair the interaction of STING and TBK1 (59, 60). These findings suggest that the STING-mediated immune defense mechanism contributes to host antiviral immune response.

Recently, it was reported that the delivery of synthetic cGAMP agonist for activation of the cGAS-STING pathway remarkably inhibited the HBV replication by inducing IFN production in the HBV-infected mouse model (48). The therapeutic drugs combined with an effective vaccine have shown high efficacy in eliminating viral hepatitis (61). As an HBV or HCV vaccine adjuvant, administration of STING agonists can induce a robust immune response *via* up-regulation of cytokines and chemokines, which may restrain tolerance in patients with chronic viral hepatitis (62, 63). Collectively, the interaction between the cGAS-STING pathway mediated innate immune response and HBV in hepatocytes and macrophages during natural infection is still elusive and controversial. Much more work is needed to investigate the molecular mechanisms underlying the role of the cGAS-STING pathway in HBV and HCV infection. These studies may provide a novel therapeutic approach for viral hepatitis.

## The cGAS-STING Pathway in Nonalcoholic Fatty Liver Disease/Nonalcoholic Steatohepatitis, and Alcoholic Liver Disease

Nonalcoholic fatty liver disease (NAFLD) is characterized by a series of diseases ranging from simple steatosis to nonalcoholic steatohepatitis (NASH), subsequent cirrhosis, and even hepatocellular carcinoma. Currently, NAFLD is increasing globally, and the prevalence of NAFLD is about 25% (64). NAFLD is becoming the most common cause of chronic liver disease and the leading cause of liver failure requiring liver transplantation in western countries (65). However, there is no safe and effective therapy for patients with NASH due to the pathogenesis of NASH not being fully understood.

**Figure 2:**
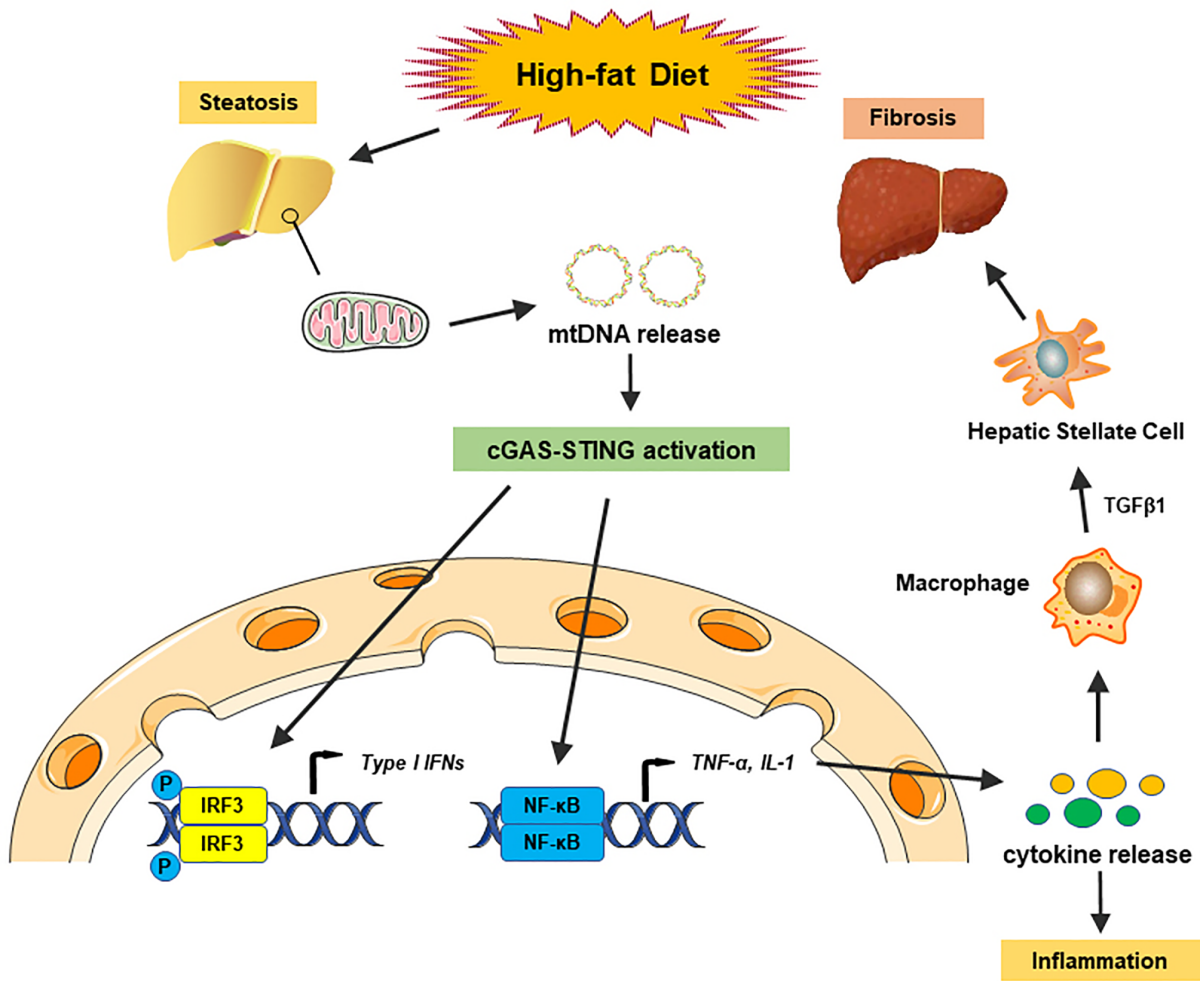
The cGAS-STING pathway in nonalcoholic fatty liver disease. A high-fat diet (HFD) causes steatosis, which induces mitochondrial stress damage in hepatocytes and subsequent releases of mitochondrial DNA (mtDNA) into the cytosol. Cytosolic mtDNA is recognized as an endogenous DAMP, which activates the cGAS-STING pathway and induces the IRF3 signaling to promote transcription of type I IFNs. Activation of the cGAS-STING pathway also induces the NF-κB signaling to produce proinflammatory cytokines, which triggers hepatic inflammatory responses. Moreover, proinflammatory cytokines activate macrophage function and produce TGF-β1, which activates hepatic stellate cells (HSCs) and promotes liver fibrosis in NASH.

It is well known that the innate immune system, especially macrophages, plays an essential role in the development of hepatic steatosis to NASH (66). During the past years, numerous reports have identified the vital role of the cGAS-STING signaling pathway in NASH progression by regulating innate immune activation. Metabolic stress, such as a high-fat diet (HFD), could activate cGAS and the STING-IRF3-mediated inflammatory response (67). By contrast, STING deficiency mitigated HFD-induced adipose tissue inflammation, obesity, insulin resistance, and glucose intolerance (67). Disruption of either STING or IRF3 significantly attenuated free fatty acid (FFA)-induced inflammatory response, lipid accumulation, and hepatocellular apoptosis through regulation of the nuclear factor κB (NF-κB) signaling pathway (68). As lipotoxicity appears to be the central driver in NASH progression by oxidative stress and ER stress (69), lipotoxic activation of TBK1, a downstream of cGAS-STING kinase, is also crucial for the control of the NASH development (70). Recently, mitochondrial DNA (mtDNA) released from injured hepatocytes has been recognized as an endogenous DAMP, which activates the cGAS-STING pathway and promotes hepatic inflammation through release of cytokines in NASH (71), suggesting that cytosolic mtDNA sensed by the cGAS-STING signaling is key to trigger innate immune response in NASH progression. Several studies have indicated that human and murine hepatocytes did not express STING protein (42, 71, 72). However, increased STING expression was observed in Kupffer cells in patients with NASH (72). Myeloid-specific STING induced TGF-β1 and activated hepatic stellate cells (HSCs), which promoted NASH progression, whereas disruption of myeloid STING alleviated hepatic inflammation, steatosis, and liver fibrosis in a mouse model of HFD or methionine and choline-deficient diet (MCD)-induced NASH (72), suggesting that activation of STING regulates macrophage function and augments hepatic lipid accumulation, profibrotic gene expression, and proinflammatory responses in NASH (**Figure 2**). Moreover, a study in liver samples from 98 patients with NAFLD revealed that STING expression in Kupffer cells and monocyte-derived macrophages (MoMFs) was correlated with hepatic inflammation and fibrosis in human NAFLD (73). These findings indicate that activation of the cGAS-STING pathway in macrophages is critical in NASH progression.

The hepatic inflammatory response has a fundamental role in NASH progression. Activation of STING induces the IRF3 and NF-κB pathways, and produces various inflammatory cytokines (74). It was reported that global knockout (KO) of IRF3 were significantly reduced liver injury, steatosis, and inflammation (75). However, another study showed that disrupted IRF3 resulted in increased insulin resistance and liver inflammation in HFD-induced NAFLD (76). Indeed, STING activated the innate immune response and contributed to the NASH progression in an NF-κB dependent manner (71). Moreover, IRF3 KO mice showed higher fasting glycemia and higher body weight (76), which was not consistent with the model of HFD-fed STING-deficient mice (71). STING might regulate glucose levels but not body weight in an IRF3-dependent manner. Further studies are needed to elucidate the underlying mechanism. Therefore, targeting STING to inhibit innate immune activation could provide a novel approach to managing NAFLD and NASH in patients.

**Figure 3:**
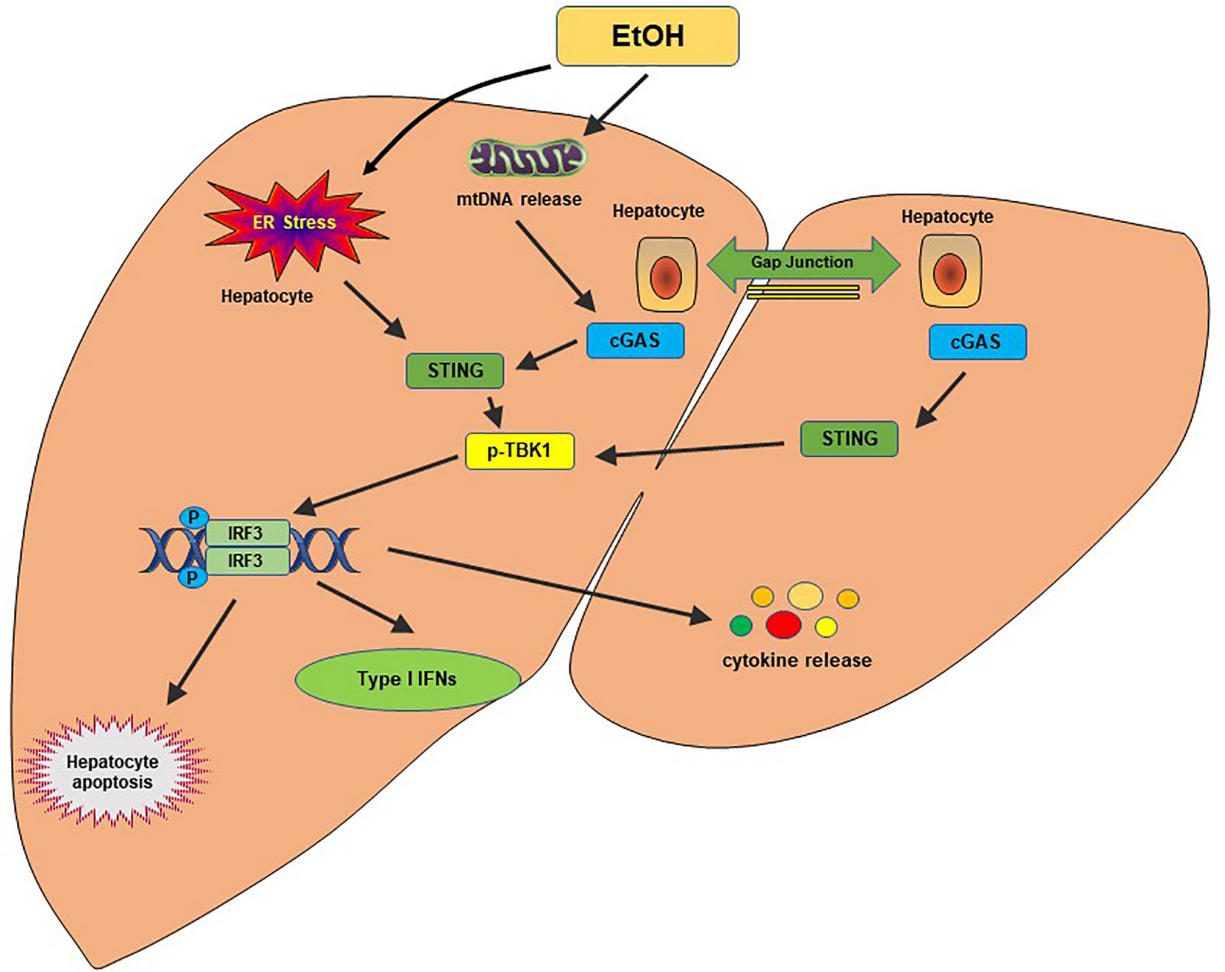
The cGAS-STING pathway in alcohol-related liver disease. Alcohol-induced ER stress and mtDNA release activate the STING pathway. STING facilitates IRF3 phosphorylation by TBK1, which results in the translocation of IRF3 into the nucleus, where it induces transcription of type I IFNs. A gap junction intracellular communication pathway between alcohol-exposed hepatocytes and the neighboring parenchyma also contributes to the IRF3 activation by cGAS. Activation of IRF3 could trigger hepatocyte apoptosis, type I IFN response and produce proinflammatory cytokines, leading to hepatic inflammation and injury.

Alcohol-related liver disease (ALD) affects more than 150 million people worldwide. It is the second most common indication for liver transplantation due to ALD-induced cirrhosis (77). Liver failure by ALD accounts for approximately half of liver cirrhosis-associated deaths in the United States (78). A previous study found that ER stress-induced IRF3 activation in the liver was associated with ER adaptor protein STING in the acute ALD model (79). IRF3 deficiency ameliorated hepatocyte apoptosis and the inflammatory responses in an ethanol-feeding mouse model (79). Alcohol-feeding remarkably increased cytoplasmic mtDNA release, resulting in activating the cGAS-IRF3 signaling (80). Activation of IRF3 by cGAS drove liver inflammation and injury in both alcohol-exposed hepatocytes and the neighboring parenchyma through a gap junction intracellular communication pathway (80). RNA-seq analysis of ALD patients showed that the cGAS-IRF3 pathway was positively associated with disease severity (80). Thus, cGAS, STING, and IRF3 are crucial determinants in the pathogenesis of ALD and potential therapeutic targets in ALD (**Figure 3**).

## The cGAS-STING Pathway in Hepatocellular Carcinoma

Hepatocellular carcinoma (HCC) is the most common primary liver cancer and is the second leading cause of cancer-related death in the world (81). Despite the availability of multiple therapeutic approaches for the early stage of HCCs, including surgical liver resection, liver transplantation, and percutaneous ablation, most patients are diagnosed at relatively advanced stages with fewer treatment options and a poor prognosis (82). Recently, cancer immunotherapy has emerged as an effective therapy for various types of cancers (83). Accumulating evidence demonstrates the vital role of the innate immune system in liver cancer immunosurveillance and immunotherapy (84). During tumorigenesis, tumor cell death and genome instability could lead to abnormal localization of genomic DNA in the cytosol and micronuclei formation (16, 85). As a solid tumor, the hypoxic microenvironment inside the HCC tumor can also induce cancer cell necrosis, which promotes the release of mitochondrial DNA (mtDNA) (86, 87). These exogenous and endogenous cytosolic DNA are subsequently recognized by the immune cells, resulting in activation of the innate immune response. Emerging studies show that cGAS also detects tumor-derived DNA, initiating antitumor immunity in some cancers (88). Indeed, the cGAS-STING pathway plays an essential role in HCC progression. It was reported that low levels of STING in tumor tissues were associated with poor prognosis in HCC patients (89). Activation of the cGAS-STING pathway augmented immune cell infiltration in HCC tissues (90). The cGAS-STING pathway members also displayed strong associations with immune markers involved in clinical stages, pathological grades, and overall survival in patients with HCC (90), suggesting that the cGAS-STING pathway members could be used as potential prognostic biomarkers in patients with HCC. In a mouse model of mutagen-induced HCC, STING deficiency reduced phosphorylated-STAT1, autophagy, and cleaved caspase 3 levels but accelerated tumor progression, with increased numbers of large tumors at advanced stages. In contrast, treatment with a cyclic dinucleotide (CDN) STING agonist promoted cell death, autophagy, and IFN responses in HCC (91). Notably, CDN treatment markedly reduced tumor size and the number of HCC in mice (91). These findings indicate STING is a promising therapeutic target for the treatment of HCC.

Immunotherapy has been rapidly expanded as a novel option in the treatment of advanced HCC. Data from the early stage of clinical trials with PD-1/PD-L1 therapy have suggested promising results with encouraging survival and safety data in HCC patients (92). While some therapeutic benefits have been reported with immune checkpoint blockade therapy, the low efficacy of immunotherapy remains a significant challenge in HCC treatment. Several studies have revealed that STING-deficient mice are less responsive to immunotherapy (93, 94). A combination treatment of cGAMP with PD-L1 inhibitor has shown a more potent antitumor effect in a xenograft model (95), indicating that stimulation of the cGAS-STING pathway may improve immunotherapeutic efficacy for the treatment of HCC. Further studies are needed to elucidate the crosstalk between the cGAS-STING and PD-1/PD-L1 pathway in antitumor immunity against HCC.

## The cGAS-STING Pathway in Liver Ischemia and Reperfusion Injury

Liver ischemia-reperfusion injury (IRI), an innate immunity-dominated local sterile inflammatory response (96), is a significant cause of hepatic dysfunction and failure in liver transplantation (97). Oxidative and ER stress are important factors in the pathogenesis of hepatic IRI. IR-induced stress activates liver macrophages (Kupffer cells) to generate reactive oxygen species (ROS), leading to sterile inflammation in the liver (98). ROS, an endogenous ‘danger’ signal released from necrotic and stressed cells, triggers toll-like receptor 4 (TLR4) or NLRP3 inflammasome-driven innate immune response in ischemic livers (98–101). ROS can induce oxidative mitochondrial damage, resulting in mtDNA leaks into the cytosol (102). The mtDNA is recognized by the DNA sensor cGAS and activates STING, which triggers an innate immune response (103). Recent studies showed that mtDNA release from hepatocytes was significantly increased during liver IRI (104). Increased mtDNA induced STING activation in macrophages after liver IRI, whereas disruption of STING reduced NLRP3 activation and proinflammatory mediators in mtDNA-stimulated macrophages from aged mice (105).

Moreover, another study showed that IR-induced stress in hepatocytes promoted cGAS expression but they did not express STING under oxidative stress conditions (106). Interestingly, cGAS global knockout (KO) mice displayed increased IR-induced liver injury compared to the wild-type or STING-deficient mice. Disruption of cGAS in hepatocytes augmented cell death and apoptosis but reduced autophagy induction in response to oxidative stress (106), suggesting that cGAS regulates hepatic autophagy in a STING-independent manner during liver IRI. Indeed, the tissue-specific roles and regulatory mechanisms of the cGAS-STING pathway remain mostly elusive. As liver macrophages, including resident Kupffer cells and infiltrated bone marrow-derived macrophages, are a major player in innate immune responses in the pathogenesis of liver IRI (98, 99, 107), it is also unclear how the cGAS-STING pathway influences the interplay between hepatocytes and innate immune cells in liver IRI. Further studies will be needed to elucidate the coordination and orchestration of these IR-stressed cells regulated by the cGAS-STING pathway.

## Concluding Remarks and Future Perspectives

It is now clear that innate immunity plays a central role in the pathogenesis of liver diseases. The innate immune response may drive the progression of liver disease and contribute to liver damage, fibrosis, cirrhosis, and even HCC. The cGAS-STING pathway functions as a direct innate immune sensor of cytosolic DNA. While self-DNA sensor cGAS can recognize cellular or tissue damages, excessive activation of the cGAS-STING pathway triggers liver inflammation and subsequent disease. Studies on the cGAS-STING pathway in liver diseases have led to a better understanding of the role of the innate immune response in the development of liver inflammation and injury. New findings involved in regulating the cGAS–STING pathway will allow us to identify the essential molecules as potential therapeutic targets for liver diseases. Indeed, the cGAS–STING pathway is a dual-edged sword. Transient activation of this pathway shows an antitumor and antiviral effect, but persistent activation may promote inflammation-driven tumorigenesis (108). cGAS-STING dependent DNA-​sensing of micronuclei in tumor cells can stimulate tumor metastasis due to chromosomal instability (109). However, tumor-derived cGAMP triggered natural killer (NK) cell response and inhibited tumor growth by activating the STING pathway (110). Although the STING agonists have shown promising results in HBV/HCV infection and HCC therapy in disease models (50, 51, 91, 111), more preclinical studies and early-stage clinical trials are needed to verify these encouraging survival and safety data.

The current research on the cGAS-STING signaling pathway in liver diseases has revealed only ‘tip of the iceberg’. Further studies on tissue-specific roles of the cGAS-STING pathway with other DNA sensing pathways in liver inflammation and injury are critical. They may provide new insights into the mechanism of therapy for liver diseases.

## Author Contributions

BK was responsible for the conception of the review. DX wrote the first draft of the manuscript and YT produced ideas for the figures. QX and BK participated in scientific discussion. All authors contributed to the article and approved the submitted version.

## Funding

This work was supported by the NIH grants R01AI139552, R21AI146742, R21AI112722, and R21AI115133.

## Conflict of Interest

The authors declare that the research was conducted in the absence of any commercial or financial relationships that could be construed as a potential conflict of interest.

## Abbreviations

ALD, alcoholic liver disease; cGAMP-AMP, cyclic guanosine monophosphate-adenosine monophosphate; cGAS, cyclic GMP-AMP synthase; DAMPs, damage-associated molecular patterns; dsDNA, double-stranded DNA; ER, endoplasmic reticulum; HCC, hepatocellular cancer; IFNs, type I interferons; IRF3, IFN regulatory factor 3; IRI, ischemia/reperfusion injury; NAFLD, nonalcoholic fatty liver disease; NASH, nonalcoholic steatohepatitis; PAMPs, pathogen-associated molecular patterns; RIG-1, retinoic acid-inducible gene I; ROS – reactive oxygen species; STING, stimulator of interferon genes; TBK1, TANK-binding kinase 1; IKK, IκB kinase; NF-κB, nuclear factor-κB.
